# From Fertilisation to Implantation in Mammalian Pregnancy—Modulation of Early Human Reproduction by the Endocannabinoid System

**DOI:** 10.3390/ph3092910

**Published:** 2010-09-02

**Authors:** Katerina N. Bambang, Tulay Karasu, Alpha Gebeh, Anthony H. Taylor, Timothy H. Marczylo, Patricia Lam, Jonathon M. Willets, Justin C. Konje

**Affiliations:** Endocannabinoid Research Group, Reproductive Sciences Section, Department of Cancer Studies and Molecular Medicine, Robert Kilpatrick Clinical Sciences Building, Leicester Royal Infirmary, Leicester LE2 7LX, UK; E-Mails: kb217@le.ac.uk (K.N.B.); tk113@le.ac.uk (T.K.); ag271@le.ac.uk (A.G.); aht3@le.ac.uk (A.H.T.); thm3@le.ac.uk (T.H.M.); pmwl1@le.ac.uk (P.L.); jmw23@le.ac.uk (J.M.W.)

**Keywords:** endocannabinoid, reproduction, fertilization, implantation

## Abstract

There is an increasing recognition that the endocannabinoid system is the crucial cytokine-hormone system regulating early human pregnancy. The synchronous development of the fertilized embryo and the endometrium to ensure timely implantation has been shown to be one of the pivotal steps to successful implantation. This development is thought to be regulated by a finely balanced relationship between various components of the endocannabinoid system in the endometrium, the embryo and the Fallopian tube. In addition, this system has also been shown to be involved in the regulation of the development and maturation of the gametes prior to fertilization. In this review, we will examine the evidence from animal and human studies to support the role of the endocannabinoid system in gametogenesis, fertilization, implantation, early pregnancy maintenance, and in immunomodulation of pregnancy. We will discuss the role of the cannabinoid receptors and the enzymes involved in the synthesis and degradation of the key endocannabinoid ligands (e.g., anandamide and 2-arachinoylglycerol) in early reproduction.

## 1. Introduction

The initial stages of pregnancy, including conception, are crucial in determining the outcome. Modern lifestyle choices have not made this easier as evident by rising rates of infertility and miscarriage [1]. It is now thought that subfertility affects 10-15% of couples and 12% of recognised pregnancies end in miscarriage [2,3]. There are obviously a number of factors influencing these outcomes including obesity, increasing maternal age, legal and illegal drug use.

Preparations of the *Cannabis sativa* plant are some of the most commonly used illegal drugs and exposure has long been associated with adverse pregnancy outcomes, including miscarriage and prematurity [1]. The main psychoactive component of the plant is Δ^9^-tetrahydrocannabinol (THC). However, chronic marijuana use is not always associated with infertility, but is associated with impaired sperm function [4,5]. What is clear is that marijuana and alcohol abuse often go ‘hand-in-hand’ and when combined, infertility problems are greatly enhanced when compared to either drug alone [6]. The discovery of cannabinoid receptors, which bind exogenous cannabinoids, fuelled a search for endogenous ligands producing similar effects; a search, which came to fruition in the 1990s with the discovery of anandamide, the first of a large family of lipid mediators able to activate cannabinoid receptors [7,8,9]. This was closely followed by the discovery that 2-arachidonoylglycerol (2-AG), an already identified compound, had cannabinoid properties [10].

Since exogenous cannabinoids are known to be harmful to pregnancy, endogenous cannabinoids may also have adverse effects. The first evidence to support this came from the observation by Wenger and colleagues that anandamide reduced luteinising hormone (LH) and prolactin levels in pregnant rats [11]. Since then, endocannabinoids have been implicated in a number of physiological processes crucial to normal conception and early gestation. The endocannabinoids, their receptors and the enzymes involved in their synthesis and degradation are together referred to as the endocannabinoid system (ECS). As the drive to develop drugs of the endocannabinoid family for various uses including anti-obesity drugs accelerates, the potential effects on the reproductive system must not be forgotten.

In this review, we will discuss the endocannabinoid system and explore its role in reproduction, focusing particularly on gametogenesis, fertilisation and development of the blastocyst and how this impacts on the establishment of a viable pregnancy.

## 2. The Endocannabinoid System

The ECS controls a plethora of physiological processes including neuronal development, inflammation, energy metabolism, cardiovascular function and reproduction to name but a few [12,13,14,15]. Arachidonoylethanolamine, anandamide (AEA) and 2-arachidonolyglycerol (2-AG) are the most studied ligands of the ECS. Anandamide is the prototypical molecule belonging to the family of lipid mediators known as *N*-acylethanolamines (NAE) and co-exists with several other similar molecules such as *N*-palmitoylethanolamine (PEA), *N-*oleolyethanolamine (OEA) and *N*-stearoylethanolamine (SEA) [13,16]. OEA and PEA display a variety of activities such as anti-inflammatory and anorexic effects which do not appear to be mediated via cannabinoid receptors, although they are thought to have an entourage effect, potentiating AEA activity [13,17,18].

### 2.1. Synthesis

NAEs are generally produced from membrane phospholipids through a two-step pathway known as the ‘transacylation-phosphodiesterase pathway’ [18,19,20]. With regards to AEA, the first step (also thought to be the rate-limiting step in its synthesis) is the *N*-acylation of phosphatidylethanolamine, which is catalyzed by Ca^2+^ dependent *N*-acyltransferase (NAT) to yield *N*-acylphosphatidylethanolamine (NAPE) [21]. An isoform of *N*-acyltransferase (iNAT) was recently cloned but this differs from NAT in that it appears to be insensitive to Ca^2+^ activation [22]. Tissue distribution of iNAT has shown that its activity was highest in the rat testis but not the rat ovary (23]. The physiological significance of this is presently unknown. 

The second step is the hydrolysis of *N*-acylphosphatidylethanolamine (NAPE) by a NAPE specific phospholipase D [NAPE-PLD) to yield AEA and phosphatidic acid [22]. NAPE-PLD belongs to the metallo-β-lactamase family and is activated by Ca^2+^ and other divalent cations such as Mg^2+,^ Co^2+,^ Mn^2+,^ Ba^2+, ^Ni^2+^ and Sr^2+^ [21,25,26].

**Figure 1:**
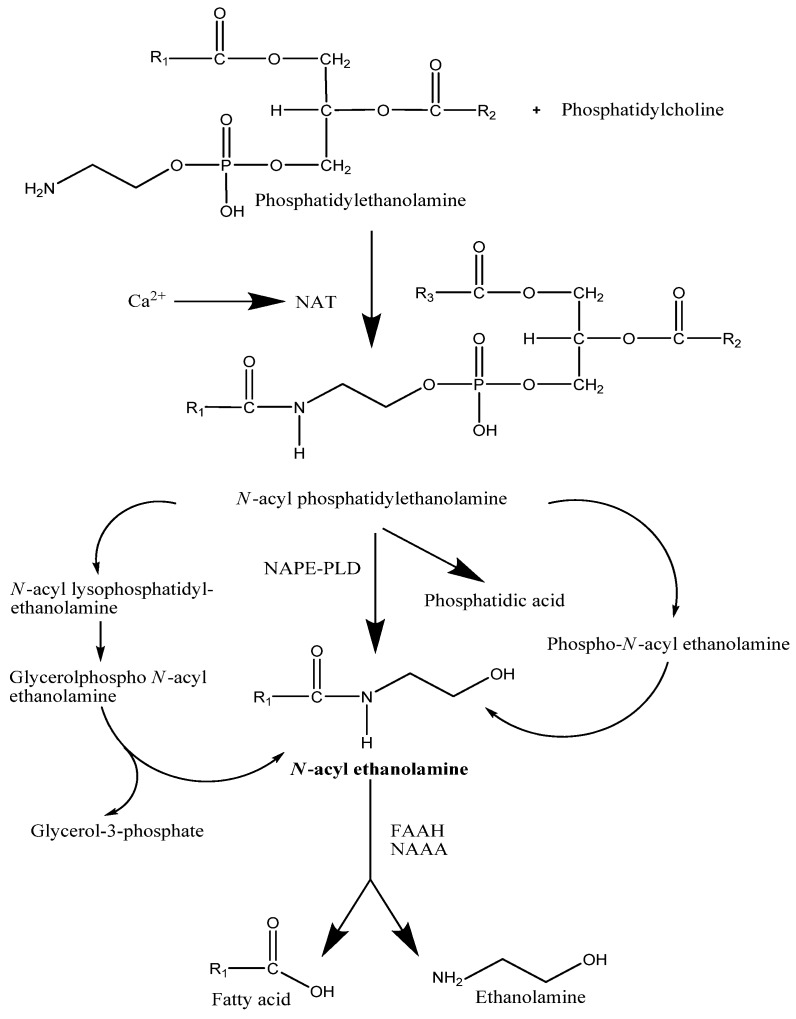
Proposed pathways for the synthesis and degradation of AEA.

**Figure 2:**
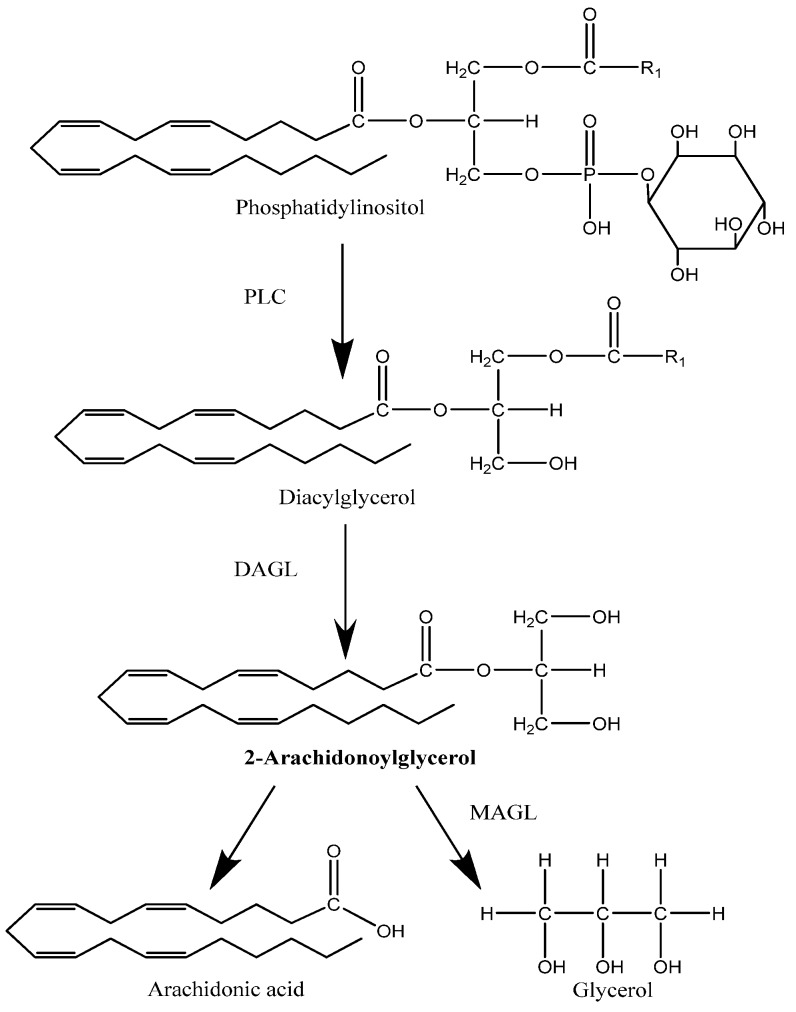
Main pathway for the synthesis and degradation of 2-arachidonoylglycerol.

Although, this is the commonly observed biosynthetic pathway for AEA, there may be at least three different metabolic pathways for the generation of AEA [26,27]. One involves sequential de-acylation of NAPE followed by cleavage of glycerophosphate to yield AEA and the second proceeds through phospholipase C mediated hydrolysis of NAPE to generate phosphoanandamide which is then de-phosphorylated by phosphatases (Figure 1) [26,27]. 2-AG, the other endocannabinoid, is also produced via a two step pathway which generates diacylglycerol (DAG) by phospholipase C (PLC) activity and subsequent hydrolysis by a membrane bound diacylglycerol lipase (DAGL) of which two isozymes (DAGLα and DAGLβ) were recently cloned (Figure 2) [28]. Both 2-AG and DAG are intermediates in several pathways, one of which leads to the generation of arachidonic acid. Thus, it may be that not all production pathways are part of the endocannabinoid system [29].

### 2.2. Degradation

Endocannabinoids are thought to be produced on demand and their actions cease following rapid transport into the cell and intracellular hydrolysis by two distinct enzyme systems. The transport of the endocannabinoid molecules into the cell remains a contentious issue as the elusive transmembrane AEA transporter (AMT) has yet to be identified [30]. Nevertheless, recent evidence points towards endocannabinoid-specific transport mechanisms via intracellular fatty acid binding protein (FABP) family members, with several of these members being involved in the promotion of AEA hydrolysis [31]. Some of the FABPs interact with the plasma membrane and thus may actually be the elusive AMT, although some fatty acids are transported via CD36 (and related molecules) suggesting that these might be the AMT [32]. In Cos7 cells stably expressing brain/neurone important FABP isoforms, only the FABP5 and FABP7 isoforms potentiated AEA hydrolysis, whereas FABP3 did not (31]. Since FABP6 has been demonstrated to be present in the rat ovary and FABP5 in the testis, FABP4 in the placenta, FABP3 in mouse spermatozoa, testis, and placenta, and a plasma membrane specific FABP in mouse spermatozoa, then there is a possibility that drugs differentially targeting these lipid carriers may have a future role in regulating local endocannabinoid concentrations, as has been discussed previously [32,33,34,35,36].

The main enzymes involved in the degradation of endocannabinoids are fatty acid amide hydrolase (FAAH) (Figure1) and monoacylglycerol lipase (MAGL) (Figure 2). FAAH-1 is a membrane bound protein found in human and animal tissues and hydrolyzes AEA into free fatty acids and ethanolamine and although a second enzyme displaying FAAH activity has recently been discovered, it is still thought that most of this hydrolysis is predominantly by FAAH-1 [38,39]. Nevertheless, there appears to be a ubiquitous distribution of FAAH-2 in peripheral tissues suggesting a significant, though as yet, unknown role [39]. In 2004, Tsuboi *et al.* characterised a third NAE hydrolyzing amidase that appears to be abundant in immune cells, specifically macrophages [38]. This enzyme, *N*-acylethanolamine-hydrolyzing acid amidase (NAAA) has a different distribution within the body compared to FAAH-1 and FAAH-2, but interestingly, shows a preference for PEA as its substrate, so whether this amidase has a fundamental role to play in inflammation remains to be seen [38].

Although 2-AG can be metabolised by FAAH *in vitro*; the main hydrolysing enzyme involved is monoacylglycerol lipase (MAGL), which appears to be responsible for about 85% of 2-AG *in vivo* degradation, which was recently confirmed using the potent MAGL inhibitor known as JZL184 [40,41,42]. To further compound the picture, two further enzymes functioning as 2-AG hydrolases, the alpha-beta hydrolases ABHD6 and ABHD12, have been identified, leading to the suggestion that, collectively, these three enzymes may control independent pools of 2-AG and therefore different signalling events [41,42]. At present none of these 2-AG degradation systems have been shown to operate in the human reproductive tract.

### 2.3. Cannabinoid Receptors and Signal Transduction

Although there are various targets for the endocannabinoids, two main specific cannabinoid receptors have been identified and characterised as seven transmembrane G-protein coupled receptors known as CB1 and CB2 [8,43,44]. These have been studied extensively both in humans and animals and although it was initially thought that CB1 was predominantly found in neuronal tissue and the CB2 in immune cells, it is now known that their body distribution is far more widespread [45,46,47,48,49] add55. These receptors are predominantly inhibitory in nature, reducing the formation of intracellular cyclic AMP, inhibiting voltage gated N, L and P/Q type Ca^2+^ channels and activating K^+^ channels and the expression of MAP kinases [50].

Besides these two prototype receptor mediated actions, endocannabinoid ligands have been shown to act independently on Ca^2+^ channels, Na^+^ channels and the background K^+^ channel, TASK1 [51,52]. More recent evidence has emerged to support the existence of other cannabinoid receptors, namely the orphan G protein coupled receptors, GPR55 and GPR119 which appear to bind a number of endocannabinoids including OEA and PEA, molecules which are both inactive at the CB1 and CB2 receptors [52,53]. These and other endocannabinoid ligands have also been found to activate peroxisome proliferator-activated receptors (PPAR), a family of nuclear receptors which exist in three isoforms: α, δ and γ known to modulate cell differentiation, lipid metabolism and inflammation [54,55]. AEA has also been shown to activate transient receptor potential vanilloid type1 (TRPV1) receptors, which are non-specific cation channels known to modulate painful stimuli such as heat and pH [56]. Evidence is now pointing towards endocannabinoids being involved in far more complicated signal transduction pathways than previously recognised. For example, retrograde AEA synaptic signalling inhibits the signal transduction processes such that secretion of several neurotransmitters, such as acetylcholine, serotonin, dopamine, and the endocannabinoids themselves, are decreased [57]. Indeed, further evidence from studies in invertebrate spermatozoa and embryos indicate that the adverse effects of cannabinergic ligands on sperm function (capacitation) and embryo development (hatching) can be negated by the presence of agonists for acetylcholine, dopamine and serotonin receptors [58]. Such data indicate that cross talk between the ECS and other neurotransmitters are essential for proper embryo development (see Section 3.2 and Section 3.5).

## 3. Gametogenesis

The processes involved in human reproduction are tightly regulated such that gametogenesis, fertilisation, blastocyst development, oviductal transport and implantation are appropriately synchronised for successful pregnancy to occur. These critical events are regulated by endocrine, paracrine and autocrine factors and of these, the ligand-receptor signalling involving endocannabinoids has been identified as one of the main networks regulating human reproduction, and thus a potential target for pharmacological intervention [59,60,61,62,63,64,65,66,67].

### 3.1. Oogenesis

Cannabinoid receptors CB1 and CB2 have been localised in human oocytes at all stages of development while the enzymes NAPE-PLD and FAAH have been localised only in the growing secondary and tertiary follicles, corpora lutea and albicans [68]. The localisation of a fully functional ECS in the human ovary suggests a role for this system in modulating ovarian function. Some evidence of this comes from studies in women undergoing controlled ovarian hyperstimulation for IVF-ET procedures, where AEA levels from follicles containing morphologically assessed “mature” oocytes (1.56+/-0.11 nM) were found to be significantly higher than in those containing “immature” oocytes (0.99+/-0.09 nM) [68]. Not only does this demonstrate that endocannabinoids may be produced in the ovary, but that they may have a role in folliculogenesis, perhaps by interacting with gonadotrophins to regulate this process. 

Various hormones, such as follicle stimulating hormone (FSH), LH and the sex steroids, estrogen and progesterone, are responsible for the changes seen in the menstrual cycle and ovulation is an important mid cycle event which occurs, following the LH surge [69,70]. The demonstration that levels of plasma AEA during the menstrual cycle in pre-menopausal women significantly correlated with FSH and estradiol imply that endocannabinoids may interact with these hormones to regulate various physiological processes in early pregnancy [70]. Moreover, plasma AEA levels are much higher around the time of ovulation than in the follicular and luteal phases of the menstrual cycle, a finding which could be further investigated for use as a biomarker in timing oocyte collection within IVF-ET programmes [70,71].

### 3.2. Spermatogenesis and Fertilisation

Spermatogenesis in mammals occurs in association with Sertoli cells, where the hormone FSH is vital. It is now acknowledged that the ECS also plays a significant role at the Sertoli cell and while the exact molecular mechanisms are complex, they are likely to involve an interplay between endocannabinoids, sex steroid hormones and gonadotrophins [67,69,73,74,75,76,77,78,79]. For example, Sertoli cells express a fully functional ECS, and FSH has been shown to modulate FAAH activity and expression [77,78,80]. This is potentially significant, as FSH indirectly affects Sertoli cell apoptosis via the ECS system and has more direct effects of sperm survival and metabolism where glycogen synthesis is increased and mitochondrial function decreased [78,81,82]. Moreover, the selective CB2 receptor agonist, JWH133 induces progression of spermatogonia towards meiosis suggesting that CB2 receptor activation promotes the meiotic progression of germ cells during spermatogenesis [78]. Additionally, 2-AG has recently been demonstrated to regulate mouse sperm motility in the tail of the epididymis in a CB1 and TRPV1-dependent manner [84]. Somewhat surprisingly, the loss of motile/live sperm by *in vitro* treatment with the CB1 antagonist SR141716, was reversed by the presence of the TRPV1 antagonist iodoresiniferotoxin, suggesting both of these two signalling pathways are associated with each other during sperm maturation in the epididymis [84]. Whether, the same applies to all species is currently unknown, although data from the boar suggests that it may [85]. Perhaps, as more data emerges, the ECS may become a potential drug target to improve abnormal sperm parameters in subfertile men.

Where successful ovulation has occurred, fertilisation usually takes place within 24-48 hours followed by the simultaneous development and tubal transport of the zygote. The ECS is expressed in bovine sperm and oviductal epithelium where *in vitro*, methanandamide (a non-hydrolysable AEA analogue) was shown to induce sperm release from oviductal epithelium via CB1 mediated signalling [86]. This effect was independent of sperm motility and the acrosome reaction suggesting it may be a mechanism involved in making sperm readily available to oocyte for fertilisation. Though this has not been demonstrated in humans, CB1 has nonetheless been demonstrated in human sperm where its activation leads to an inhibition of sperm motility and the acrosome reaction; mechanisms which, no doubt affect the ability of sperm to fertilise an oocyte [87,88]. At the same time, activation of the TRPV1 receptor inhibits spontaneous capacitation and progesterone-induced acrosome reactions in the human and boar sperm, suggesting that the autocrine regulation of sperm function is important, at least in the human and pig [85,89]. This autocrine regulation of sperm function by the ECS is very similar to the autocrine regulation of sperm function demonstrated by cholinergic signalling in that local synthesis and receptors for both the cannabinergic and cholinergic co-localise, but not identical in other respects, because progesterone-induced acrosomal reactions by acetylcholine receptor antagonists in ejaculated human sperm were not inhibited [92].

### 3.3. Blastocyst Development

After the oocyte is fertilised, it then develops into the multi-cellular blastocyst, consisting of the trophoblast (which becomes the placenta) and the inner cell mass (which forms the pre-implantation embryo). NAPE-PLD, CB1, CB2 are expressed in the early stages of mouse pre-implantation embryo with CB1 detected from the four cell stage as opposed to CB2 found as early as the one cell stage. The earliest stage at which FAAH and NAPE-PLD are expressed is the 2-cell stage [66,93,94]. However, FAAH levels are significantly up regulated as development proceeds to the blastocyst stage. In homozygous FAAH knockout mice, embryos recovered from day 3 of pregnancy showed delayed and asynchronous development compared to that of wild type mice suggesting that the endocannabinoid system is intimately involved in modulating early blastocyst development and that a low level of anandamide is beneficial whereas high levels are detrimental to blastocyst development [66,93,94]. Furthermore, Paria *et al.* have demonstrated that while almost all embryos on day 4 of pregnancy from wild type mice were blastocysts, only 60-70% of those from CB1 knockout, CB2 knockout and CB1/CB2 double knockout embryos were at a similar stage [94]. Similar data on the direct effects of endocannabinoids on human embryo/blastocysts development are lacking, however, both the trophoblast and inner cell mass of mouse blastocyst produce a “FAAH activator” which significantly enhances the activity of FAAH in the uterus perhaps an inbuilt mechanism to protect the developing blastocyst from the noxious effects of endocannabinoids [95]. Human blastocysts may also locally regulate endocannabinoids by such a mechanism though that remains to be proven but, the observation that low levels of anandamide and high levels of FAAH in peripheral mononuclear cells occur during the putative “implantation window” in humans suggests a critical role for the ECS in regulating blastocyst development and implantation [96,97].

### 3.4. Oviductal Transport

Where embryo development is normal, CB1 receptor signalling plays a crucial role in their oviductal transport. CB1, CB1/CB2 double knockout mice retain a large number of embryos in the oviduct on day 4 of pregnancy; most of which were in the morula or blastocyst stage in contrast to CB2 knockout or wild type mice where none was retained [63,95]. In addition, wild type mice when treated with the CB1 receptor antagonist (SR141716) retained embryos in their oviducts; an effect not seen with the CB2 antagonist (SR14528) providing evidence that oviductal transport is delayed in CB1 deficient mice [63,95]. These results are not surprising given that a balance between anandamide synthesis and degradation creates a locally appropriate “anandamide tone” which permits normal embryo development and oviductal transport [62]. While these studies support a role for the ECS in normal oviductal function, evidence from human studies to support this is limited, but nonetheless, CB1 expression has been demonstrated in human Fallopian tube smooth muscle both in the follicular and luteal phases of the menstrual cycle and in ectopic pregnancy [97]. In the latter group of patients, CB1 mRNA expression in Fallopian tubes was lower compared to non-pregnant controls; an observation that lends support to a role for the ECS in modulating human embryo transport [97]. However, questions relating to the role of other ligands (such as OEA, PEA, SEA and 2-AG) and the receptors of the ECS in regulating embryo tubal transport, remain to be elucidated.

### 3.5. Implantation

Several studies have confirmed a crucial role for the endocannabinoid system in implantation where the trophectoderm of the blastocyst interacts with the uterine epithelium and leads to apposition and adhesion to the endometrium followed by trophoblast invasion [98,99,100,101]. This process of implantation occurs at a critical time following fertilisation, also known as the window of uterine receptivity for implantation; a process, which alongside blastocyst activation is coordinated, by estrogen and progesterone [94].

The endocannabinoid system has been extensively studied in animals where it has been shown that low levels of AEA and CB1 receptor expression are important for mouse implantation. AEA regulates blastocyst activation and implantation by activating ERK and Ca^2+^ signalling via CB1 receptors [94]. The different downstream signalling pathways are dependent on the concentration of AEA such that low AEA levels are found to activate ERK signalling whereas high AEA levels inhibit calcium mobilisation. CB1 expression is lower in activated blastocyst and higher in dormant blastocyst therefore coordinated down regulation of blastocyst CB1 receptor expression and uterine AEA levels are important for a successful implantation [94]. 

At the implantation site in the mouse uterus, low NAPE-PLD activity was measured and at the inter-implantation site, the enzyme activity was found to be increased leading to elevated AEA levels [102]. Furthermore, elevated levels of FAAH were found at the implantation site and lower levels at the inter-implantation site demonstrating the inverse relationship between anandamide and FAAH in mouse uterus [102,103,104].

As described above, mouse blastocysts are also able to contribute to the development of an environment for successful implantation through the release of an uncharacterised lipid “FAAH activator” [105]. Previous studies have determined the importance of AEA levels in the regulation of the preimplantation embryo and have shown that exposure to high AEA levels can be toxic to the implanting blastocyst [106,107]. Turco *et al.* demonstrated that high AEA concentrations (28nM) cause cell apoptosis and inhibit cell proliferation in sheep blastocysts via the CB1 receptor which correlates with murine studies which revealed that exposure to high levels of THC inhibits blastocyst formation, zonal hatching and trophoblastic growth [65,104,106,108]. Furthermore, adverse pregnancy outcomes such as retarded embryo development with fetal abnormalities and teratological malformations have been demonstrated following THC exposure [109].

Experiments on FAAH deficient mice have provided insight into the role of this enzyme in regulating AEA levels. FAAH deficiency leading to enhanced AEA signalling and thus impairs normal embryo development and timely implantation [93]. Studies have shown that even a short delay in implantation causes defective feto-placental development and poor pregnancy outcome [110]. The importance of the ECS has also been demonstrated in human studies where plasma AEA levels and FAAH activity in lymphocytes were shown to fluctuate during the menstrual cycle [70,97]. Maximal plasma levels of FAAH were found during the ‘window of implantation’ and AEA levels were markedly reduced during this time period [111]. It is interesting that lymphocytic FAAH activity fluctuates with progesterone levels throughout the cycle, which may be explained by the finding that FAAH is up regulated by progesterone [111,112,113]. We have recently shown, in a study of the ECS during the menstrual cycle that the highest plasma AEA level was at the time of ovulation and the lowest level in the luteal phase. In addition, there was a positive correlation between plasma and serum AEA, estradiol and FSH but not progesterone levels [70]. 

Women undergoing *in vitro* fertilisation have a successful implantation when low serum AEA levels and FAAH activity are measured at 6 weeks gestation [112]. Our group confirmed that the presence of low plasma AEA levels at 6 weeks gestation in women was a sign of a successful IVF- embryo transfer; higher AEA levels were also found in women with a threatened miscarriage who subsequently experience an early pregnancy loss [97,113]. This observation correlates with the findings that decreased FAAH activity in peripheral lymphocytes is present in women with pregnancy failure [114]. Trabucco *et al.* investigated placental tissue from women after a spontaneous miscarriage and showed low FAAH expression and increased CB1 expression [115]. Thus it seems to be that high FAAH levels and consequently low AEA levels are required for a successful implantation in humans; findings which could potentially be used to predict pregnancy outcome. 

Research to date has underlined a possible important role for the endocannabinoid system in regulating the events surrounding implantation and the maintenance of early pregnancy, the evidence suggests that any dysregulation of this tightly controlled system can lead to pregnancy failure.

### 3.6. Early Pregnancy Maintenance

The various components of the endocannabinoid system are expressed in the first trimester placenta. FAAH expression and activity is important in early placental development and its expression has been identified in 1^st^ trimester trophoblast tissue together with CB1 and CB2 receptors [116,117,118,119]. FAAH mRNA levels in 1^st^ trimester trophoblast tissue appear to be regulated during gestation with a peak at 11 weeks thus ensuring that there are low levels of AEA required for successful early placental development and protection of the developing fetus [120]. Similar results have been shown in another study where women with a normal pregnancy had peak plasma FAAH activity at 9-10 weeks gestation [117]. Our group also showed an increase in FAAH expression in trophoblastic tissue towards the end of the 1^st^ trimester followed by a decline between 10 and 12 weeks gestation [116]. It is of note that very low or absent FAAH expression together with high CB1 levels are found in placental tissue of women with a spontaneous miscarriage whereas high levels of FAAH expression are identified in placental tissue of women with a normal pregnancy [115]. This also underlines the significance of high FAAH levels at the maternal fetal interface for the development of a normal pregnancy. 

NAPE-PLD has been identified in placental tissue and it is thought that its presence enables the placenta to synthesise AEA [117,120]. Interestingly high NAPE-PLD mRNA expression is observed in normal placental tissue of women undergoing a termination of pregnancy compared to women with a spontaneous miscarriage. A possible explanation may be that placental NAPE-PLD and FAAH establish the right “anandamide tone” necessary for the fetal development [115].

It has already been shown that high AEA levels induce cell death, so our group investigated the effect of high AEA levels on trophoblast tissue and how this can lead to miscarriage [116]. The human choriocarcinoma cell line BeWo, a good model for the human first trimester trophoblast was treated with different AEA concentrations. Following treatment with excess of 3 µM AEA, inhibition of cell proliferation via the CB2 receptor was seen [116]. The anti-proliferative effect of high AEA concentrations seen in these experiments provides an explanation of how elevated plasma AEA levels may cause an increased risk of first trimester miscarriage. In successful pregnancies, plasma AEA levels are low in the luteal phase and the 1^st^ trimester and fall further in the 2^nd^ and 3^rd^ trimester [120]. 

### 3.7. Endocannabinoids, Immunomodulation and Pregnancy

To date, research in the role of the ECS in reproductive functions has primarily been searching for a direct link but certain key studies suggest there may be a more complex relationship involving key immune cells [60,113,116]. How the antigenically separate blastocyst implants and develops in the maternal uterus is still incompletely understood. During early pregnancy, there is a plethora of immunocytes present in the decidua, the main ones being natural killer (NK) cells, macrophages and T helper cells [121,122]. Murine studies have suggested that type-2 T helper cells (Th2) release cytokines such as the interleukins, IL4, IL10 and leukaemia inhibitory factor (LIF) known to favour implantation but also inhibit type-1 helper T cell cytokines (IL2, IL12, IFN-γ), which are thought to be responsible for rejection of the allograft [122,123,124].

Immune cells express both CB1 and CB2 receptors with AEA biosynthesis and degradation occurring in both macrophages and leukocytes [124,125,126,127]. Endocannabinoids have not only been shown to block Th1 cytokines but to also support production of Th2 cytokines [128,129,130]. It is of interest to note that Th2 cytokines, such as IL4 have been shown to stimulate FAAH activity, suggesting a possible synergistic role in reducing the level of AEA at the implantation site [130]. AEA and PEA have also been shown to inhibit, via CB2 receptors, the production of inflammatory mediators in murine RAW264.7 macrophages [130]. Most research in this area has focused on peripheral actions of endocannabinoids but the findings are highly suggestive of a possible role in localised reproductive events, which remains to be elucidated.

## 4. Conclusions

Although there is now an overwhelming body of evidence implicating the endocannabinoid system in reproductive functions and this makes it an attractive target for potential pharmacological modulation, difficulties arise with interventions because of the natural complexity of the system and the fact that numerous as yet uncharacterised molecules involved in the ECS such as the putative AMT make it difficult to predict effects. Furthermore, it is almost impossible to identify a specific focus for therapeutic manipulation when each ligand is a substrate for so many distinct receptor families. There have been significant advances made in the development of synthetic drugs including receptor agonists and antagonists, drugs inhibiting FAAH activity and drugs blocking endocannabinoid transport but such pharmaceuticals need to be examined very carefully, especially with reference to reproduction. For example, Rimonabant, a CB1 receptor antagonist, was designed as an anti-obesity drug following on from animal studies showing that blocking the CB1 receptor would reduce food intake and stimulate weight loss [131,132,133]. Unfortunately, the high incidence of side effects resulted in its withdrawal from the market. There has been a prolonged debate as to whether these adverse reactions were attributable to the drug or not but nevertheless it has highlighted the fact that with a signalling system as sophisticated and far reaching as that of the endocannabinoids, it will be difficult to design a drug which has the specificity required for a particular effect especially when the potential long term side effects of such drugs have not been elaborated. This review has examined the relevance of the endocannabinoid system from gametogenesis to early pregnancy and briefly highlighted the possible implications such drugs could have on human reproduction. The best way to really make a positive step forward in the design of therapeutics that affect endocannabinoid signalling is to concentrate on the complete characterisation of the system in establishing and maintaining pregnancy.
